# Interpretable multimodal deep learning for real-time pan-tissue pan-disease pathology search on social media

**DOI:** 10.1038/s41379-020-0540-1

**Published:** 2020-05-28

**Authors:** Andrew J. Schaumberg, Wendy C. Juarez-Nicanor, Sarah J. Choudhury, Laura G. Pastrián, Bobbi S. Pritt, Mario Prieto Pozuelo, Ricardo Sotillo Sánchez, Khanh Ho, Nusrat Zahra, Betul Duygu Sener, Stephen Yip, Bin Xu, Srinivas Rao Annavarapu, Aurélien Morini, Karra A. Jones, Kathia Rosado-Orozco, Sanjay Mukhopadhyay, Carlos Miguel, Hongyu Yang, Yale Rosen, Rola H. Ali, Olaleke O. Folaranmi, Jerad M. Gardner, Corina Rusu, Celina Stayerman, John Gross, Dauda E. Suleiman, S. Joseph Sirintrapun, Mariam Aly, Thomas J. Fuchs

**Affiliations:** 1 Memorial Sloan Kettering Cancer Center and the Tri-Institutional Training Program in Computational Biology and Medicine, New York, NY USA; 2 Weill Cornell Graduate School of Medical Sciences, New York, NY USA; 3 Weill Cornell High School Science Immersion Program, New York, NY USA; 4 Manhattan/Hunter Science High School, New York, NY USA; 5 Department of Pathology, Hospital Universitario La Paz, Madrid, Spain; 6 Department of Laboratory Medicine and Pathology, Mayo Clinic, Minneapolis, MN USA; 7 Laboratorio de Dianas Terapéuticas, Hospital Universitario HM Sanchinarro, Madrid, Spain; 8 Departamento de Patología, Virgen de Altagracia Hospital, Manzanares, Spain; 9 Département de Pathologie, Centre Hospitalier de Mouscron, Manzanares, Belgium; 10 Department of Pathology, Allama Iqbal Medical College, Lahore, Pakistan; 11 Department of Pathology, Konya Training and Research Hospital, Konya, Turkey; 12 Department of Pathology, BC Cancer, Vancouver, BC Canada; 13 Department of Pathology, Sunnybrook Health Sciences Centre, Toronto, ON Canada; 14 Department of Cellular Pathology, Royal Victoria Infirmary, England, UK; 15 Faculté de médecine de Créteil, Université Paris Est Créteil, Créteil, France; 16 Department of Pathology, University of Iowa, Iowa City, IA USA; 17 HRP Labs, San Juan, PR USA; 18 Department of Pathology, Cleveland Clinic, Cleveland, OH USA; 19 Department of Pathology, Centro Médico de Asturias, Oviedo, Spain; 20 Department of Pathology, St Vincent Evansville Hospital, Evansville, IN USA; 21 Department of Pathology, SUNY Downstate Medical Center, New York, NY USA; 22 Faculty of Medicine, Kuwait University, Kuwait City, Kuwait; 23 Department of Pathology, University of Ilorin Teaching Hospital, Ilorin, Nigeria; 24 Department of Pathology, University of Arkansas for Medical Sciences, Little Rock, AK USA; 25 Department of Pathology, Augusta Hospital, Bochum, Germany; 26 Laboratorio TechniPath, San Pedro Sula, Honduras; 27 Bone and Soft Tissue and Surgical Pathology, Mayo Clinic, Rochester, MN USA; 28 Department of Histopathology, Abubakar Tafawa Balewa University Teaching Hospital, Bauchi, Nigeria; 29 Department of Pathology, Memorial Sloan Kettering Cancer Center, New York, NY USA; 30 Department of Psychology, Columbia University, New York, NY USA; 31 Affiliate Member of Zuckerman Mind Brain Behavior Institute, Columbia University, New York, NY USA

**Keywords:** Bioinformatics, Diagnostic markers, Pathology

## Abstract

Pathologists are responsible for rapidly providing a diagnosis on critical health issues. Challenging cases benefit from additional opinions of pathologist colleagues. In addition to on-site colleagues, there is an active worldwide community of pathologists on social media for complementary opinions. Such access to pathologists worldwide has the capacity to improve diagnostic accuracy and generate broader consensus on next steps in patient care. From Twitter we curate 13,626 images from 6,351 tweets from 25 pathologists from 13 countries. We supplement the Twitter data with 113,161 images from 1,074,484 PubMed articles. We develop machine learning and deep learning models to (i) accurately identify histopathology stains, (ii) discriminate between tissues, and (iii) differentiate disease states. Area Under Receiver Operating Characteristic (AUROC) is 0.805–0.996 for these tasks. We repurpose the disease classifier to search for similar disease states given an image and clinical covariates. We report precision@*k* = 1 = 0.7618 ± 0.0018 (chance 0.397 ± 0.004, mean ±stdev ). The classifiers find that texture and tissue are important clinico-visual features of disease. Deep features trained only on natural images (e.g., cats and dogs) substantially improved search performance, while pathology-specific deep features and cell nuclei features further improved search to a lesser extent. We implement a social media bot (@pathobot on Twitter) to use the trained classifiers to aid pathologists in obtaining real-time feedback on challenging cases. If a social media post containing pathology text and images mentions the bot, the bot generates quantitative predictions of disease state (normal/artifact/infection/injury/nontumor, preneoplastic/benign/low-grade-malignant-potential, or malignant) and lists similar cases across social media and PubMed. Our project has become a globally distributed expert system that facilitates pathological diagnosis and brings expertise to underserved regions or hospitals with less expertise in a particular disease. This is the first pan-tissue pan-disease (i.e., from infection to malignancy) method for prediction and search on social media, and the first pathology study prospectively tested in public on social media. We will share data through http://pathobotology.org. We expect our project to cultivate a more connected world of physicians and improve patient care worldwide.

## Introduction

The United Nations’ Sustainable Development Goal 3: Good Health and Well-Being suggests that it is essential to “ensure healthy lives and promote well-being for all at all ages” [1]. In the furtherance of this goal, it is suggested to “[s]ubstantially increase [...] the recruitment, development, training, and retention of the health workforce in developing countries” to universally achieve “access to quality essential health care services” [1]. We therefore take connecting pathologists worldwide to be important. Indeed, Nix et al. [2] find pathologists in developing countries (e.g., India, Brazil, and Pakistan) frequently use social media, and 220/1014 (22%) of the posts they analyzed involved “asking for opinions on diagnosis”. The use of social media by pathologists occurs worldwide for both challenging cases and education [3–5]. This suggests social media can facilitate global collaborations among pathologists for novel discoveries [6]. We expand on these approaches by combining (i) real-time machine learning with (ii) expert pathologist opinions via social media to facilitate (i) search for similar cases and (ii) pathological diagnosis by sharing expertise on a particular disease, often with underserved hospitals.

For machine learning to work in general practice, it must be trained on data (i) of sufficient diversity to represent the true variability of what is observed (ii) in a sufficiently realistic setting that may differ from tightly controlled experimental conditions [7]. We therefore (i) collaborate with pathologists worldwide where we (ii) use for training the images that these pathologists share to obtain opinions, which are often histopathology microscopy pictures from a smartphone. We did not observe many images from whole-slide scanners, which at a global scale have been adopted slowly, due in part to cost and complexities of digital pathology workflows [8, 9].

For machine learning to work accurately, it must be trained on a sufficiently large dataset. Our first aim is therefore to curate a large dataset of pathology images for training a machine learning classifier. This is important because in other machine learning domains, e.g., natural vision tasks, datasets of millions of images are often used to train and benchmark, e.g., ImageNet [10] or CIFAR-10 [11]. Transfer learning allows limited repurposing of these classifiers for other domains, e.g., pathology [12–15]. Indeed, we [16] are among many who start in computational pathology [17] with deep neural networks pretrained on ImageNet [18–20], and we do so here.

However, computational pathology datasets annotated for supervised learning are often much smaller than millions of images. For example, there are only 32 cases in the training data for a Medical Image Computing and Computer Assisted Intervention challenge (available at http://miccai.cloudapp.net/competitions/82) for distinguishing brain cancer subtypes, and this includes both pathology and radiology images. Other studies are larger, such as the TUmor Proliferation Assessment Challenge (TUPAC16) dataset of 821 cases [21] all 821 cases being whole slide images from The Cancer Genome Atlas (TCGA) (http://cancergenome.nih.gov/). TCGA has tens of thousands of whole slide images available in total, but these images are only hematoxylin and eosin (H&E) stained slides, and do not represent nonneoplastic lesions such as infections, which are clinically important to correctly diagnose [22]. The main limitation is that obtaining annotations from a pathologist is difficult due to outstanding clinical service obligations, which prevented our earlier efforts from scaling up [23]. We overcome this limitation by curating a large and diverse dataset of 13,626 images from Twitter and 113,161 images from PubMed, where text annotations came from social media post text, hashtags, article titles, abstracts, and/or figure captions.

Equipped with our large dataset, we then address our second main aim, which is to utilize machine learning trained on this dataset to facilitate prospective disease state predictions and search from pathologists in real time on social media. To that end, we capitalize on a common and systematic approach to diagnosis in which a disease is in one of three classes [22]. Specifically, we use machine learning on pathology images from social media and PubMed to classify images into one of three disease states: nontumor (e.g., normal, artifact (Fig. S1), injury, infection, or nontumor), low grade (e.g., preneoplastic, benign, or low-grade malignant potential), or malignant.

**Fig. 1:**
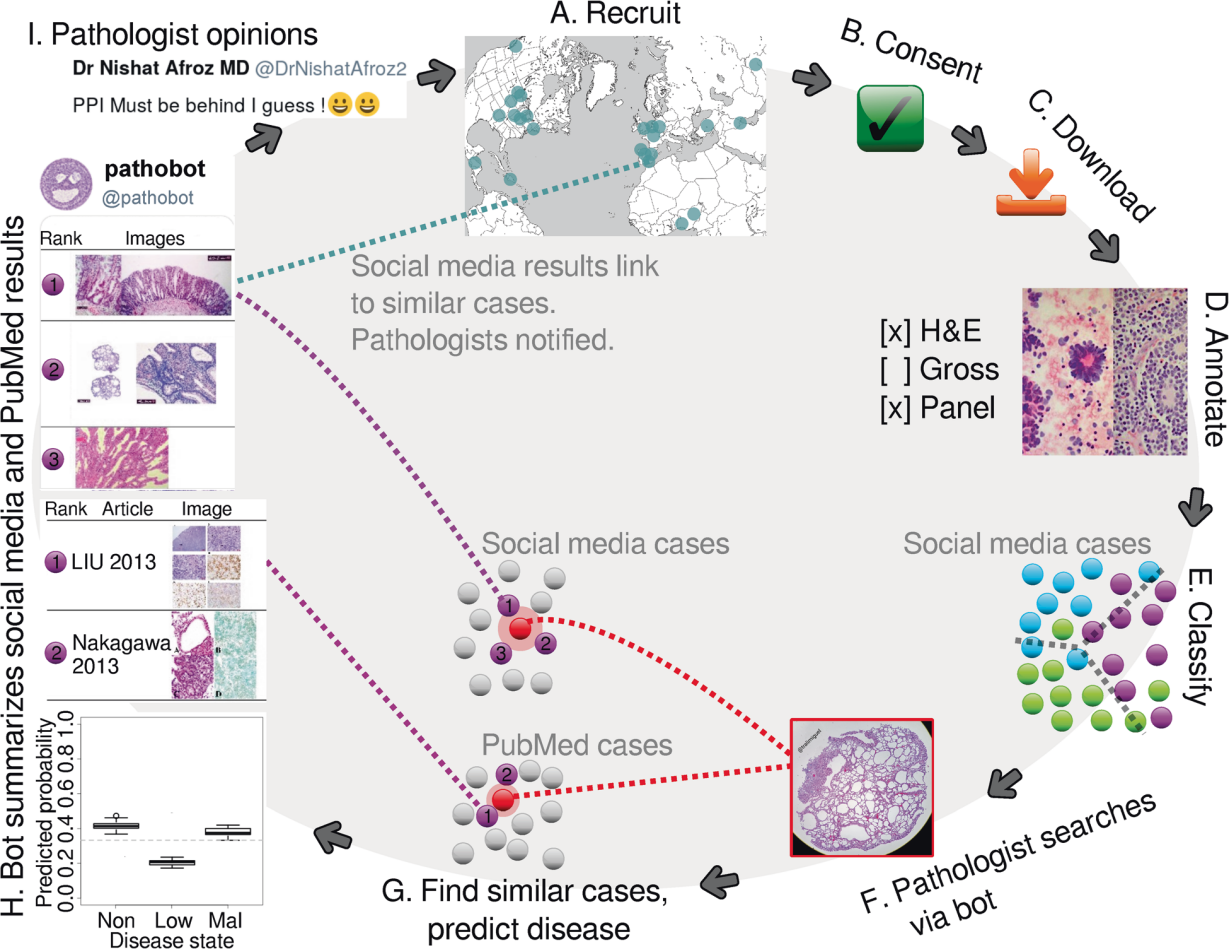
Graphical summary. Pathologists are recruited worldwide (**A**). If a pathologist consents to having their images used (**B**), we download those images (**C**) and manually annotate them (**D**). Next, we train a Random Forest classifier to predict image characteristics, e.g., disease state (**E**). This classifier is used to predict disease and search. If a pathologist posts a case to social media and mentions @pathobot (**F**), our bot will use the post’s text and images to find similar cases on social media and PubMed (**G**). The bot then posts summaries and notifies pathologists with similar cases (**H**). Pathologists discuss the results (**I**), and some also decide to share their cases with us, initiating the cycle again (**A**). “Procedure overview” in the supplement explains further (Section S5.4).

We then implement a social media bot that in real time applies our machine learning classifiers in response to pathologists on social media to (i) search for similar cases, (ii) provide quantitative predictions of disease states, and (iii) encourage discussion (Fig. 1). When this bot links to a similar case, the pathologist who shared that case is notified. The ensuing discussions among pathologists are more informative and context-specific than a computational prediction. For instance, to make a diagnosis of Kaposi’s sarcoma, first-world countries have access to an HHV8 histopathology stain, but a pathologist in a developing country may instead be advised to check patient history of HIV because the HHV8 stain is prohibitively expensive. Obviously, a computational prediction of cancer/non-cancer is far less helpful than what humans do: discuss.

In order for machine learning approaches to be useful in a clinical setting, it is critical that these approaches be interpretable and undergo rigorous prospective testing [24]. Furthermore, these approaches need to be accompanied by quantified measures of prediction uncertainty [25]. It may be argued whenever human life is at risk—(i) interpretability, (ii) uncertainty quantification, and (iii) prospective testing are essential—whether the context is medicine or self-driving cars [26, 27]. Our social media bot and methods are the first in computational pathology to meet all of these criteria in that (i) we provide multiple levels of interpretability (e.g., Random Forest feature importance and deep learning activation heatmaps), (ii) we statistically quantify prediction uncertainty using ensemble methods, and (iii) we prospectively test in full public view on social media. Concretely, this means (i) a pathologist can interpret what concepts the machine learning finds to be diagnostic in general or what parts of a particular image suggest a specific disease state, (ii) statistical significance, confidence intervals, or boxplots of computational predictions are presented to a pathologist for assessment (e.g., the boxplot in Fig. 1H lower left), and (iii) in real time a pathologist can interact with our social media bot and method to appraise performance on a case-by-case basis, as well as evaluate the public history of pathologist–bot interactions on social media.

## Materials and methods

This study was approved by the Institutional Review Board at Memorial Sloan Kettering Cancer Center.

### Social media data

From Twitter we curate 13,626 images from 6,351 tweets from 25 pathologists from 13 countries. We chose Twitter primarily for its brevity, i.e., one Tweet is at most 280 characters, so we did not expect to need complicated text processing logic to parse tissues or diagnoses. Written permission to download and use the data was obtained from each collaborating pathologist. One pathologist publicly declared their data free to use, so we use these data with acknowledgement. One pathologist donated his glass slide library to another pathologist, and the receiving pathologist shared some received cases on social media, which we treat as belonging to the receiving pathologist. Images are annotated with their tweet text and replies. We use these data for supervised learning.

### PubMed data

**Fig. 2:**
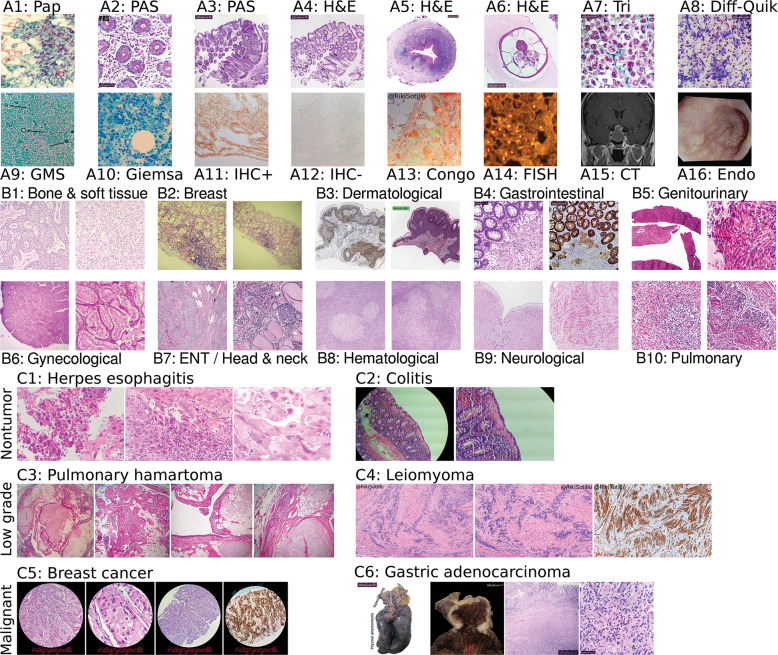
Technique, tissue, and disease diversity. Panel set **A** shows diverse techniques in our data. Initials indicate author owning image. **A1** RSS: papanicolaou stain. **A2** LGP: periodic acid–Schiff (PAS) stain, glycogen in pink. **A3** LGP: PAS stain, lower magnification. **A4** LGP: H&E stain c.f. Panel A3. **A5** LGP: H&E stain, human appendix, including parasite *Enterobius vermicularis* (c.f. Fig. S2). **A6** LGP: Higher magnification *E. vermicularis* c.f. Panel A5. **A7** LGP: Gömöri trichrome, collagen in green. **A8** LGP: Diff-quik stain, for cytology. **A9** RSS: GMS stain (“Intra-stain diversity” in supplement details variants, Section S5.3.1), fungi black. **A10** MPP: Giemsa stain. **A11** AM: immunohistochemistry (IHC) stain, positive result. **A12** AM: IHC stain, negative result. **A13** RSS: Congo red, polarized light, plaques showing green birefringence. **A14** MPP: fluorescence in situ hybridization (FISH) indicating breast cancer *Her2* heterogeneity. **A15** SY: head computed tomography (CT) scan. **A16** LGP: esophageal endoscopy. In panel set **B** we show differing morphologies for all ten histopathological tissue types on Twitter. **B1** CS: bone and soft tissue. We include cardiac here. **B2** KH: breast. **B3** RSS: dermatological. **B4** LGP: gastrointestinal. **B5** OOF: genitourinary. **B6** MPP: gynecological. **B7** BX: otorhinolaryngological a.k.a. head and neck. We include ocular, oral, and endocrine here. **B8** CS: hematological, e.g., lymph node. **B9** SY: neurological. **B10** SM: pulmonary. In panel set **C** we show the three disease states we use: nontumor, low grade, and malignant. **C1** MPP: nontumor disease, i.e., herpes esophagitis with Cowdry A inclusions. **C2** KH: nontumor disease, i.e., collagenous colitis showing thickened irregular subepithelial collagen table with entrapped fibroblasts, vessels, and inflammatory cells. **C3** AM: low grade, i.e., pulmonary hamartoma showing entrapped clefts lined by respiratory epithelium. **C4** RSS: low grade, i.e., leiomyoma showing nuclear palisading. We show IHC completeness but it is not included for machine learning. **C5** BDS: malignant, i.e., breast cancer with apocrine differentiation. **C6** LGP: malignant, i.e., relapsed gastric adenocarcinoma with diffuse growth throughout the anastomosis and colon. Gross sections (e.g., Fig. S3) shown for completeness but not used.

To represent PubMed data, we download the PubMed Central “Open Access Subset” of 1,074,484 articles. We first trained a classifier to distinguish H&E images from all others on social media (Figs. 2, S4, and S5), then used the classifier to identify PubMed articles that have at least one H&E figure. From the identified 30,585 articles we retain 113,161 H&E images to comprise our PubMed dataset. Images are annotated with figure caption, article abstract, and article title. This expanded dataset may contain disease that is too rare to be represented in social media data.

### Image processing

We manually curate all social media images, separating pathology from non-pathology images. “Defining an acceptable pathology image” (Section S5.11) details this distinction in the supplement (Fig. S4). Some pathologists use our Integrated Pathology Annotator tool to browse their data and manually curate the annotations for their cases (Figs. S6, S7). We retain non-pathology data publicly posted by consenting pathologists that cannot be publicly distributed to enable building a machine learning classifier that can reliably distinguish pathology from non-pathology images.

### Text processing

“Text data overview” (Section S5.5) in the supplement discusses our text processing to derive ground truth from social media posts (Fig. S8). We use hashtags, e.g., #dermpath and #cancer, as labels for supervised learning. We process the text of the tweet and the replies, searching for terms that indicate tissue type or disease state. For instance, “ovarian” typically indicates gynecological pathology, while “carcinoma in situ” typically indicates low-grade disease (specifically, preneoplastic disease in our low-grade disease state category). Our text processing algorithm (Fig. S8) is the result of author consensus.

### Random Forest classifier

**Fig. 3:**
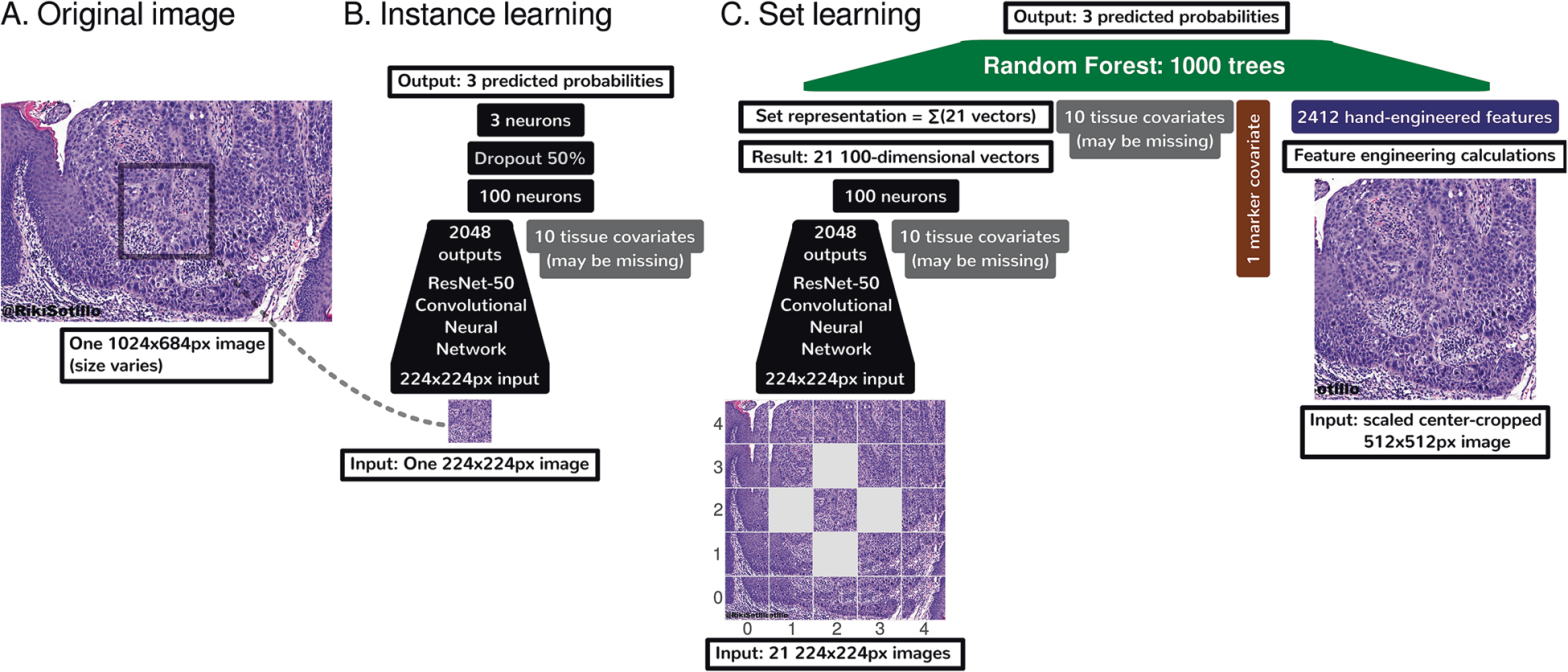
Deep learning methods summary. **A** An overall input image may be of any size, but must be at least 512 × 512 pixels (px). **B** We use a ResNet-50 [29] deep convolutional neural network to learn to predict disease state (nontumor, low grade, or malignant) on the basis of a small 224 × 224 px patch. This small size is required to fit the ResNet-50 and image batches in limited GPU memory. **C** For set learning, this network transforms each of the 21 patches sampled evenly from the image in a grid to a 100-dimensional vector. These 21 patches span the overall input image entirely. For instance, if the overall input image is especially wide, the 21 patches will overlap less in the *X* dimension. The ResNet-50 converts these 21 patches to 21 vectors. These 21 vectors are summed to represent the overall image, regardless of the original image’s size, which may vary. This sum vector is concatenated with tissue covariates (which may be missing for some images), marker mention covariate, and hand-engineered features. A Random Forest then learns to predict disease state on this concatenation that encodes (i) task-agnostic hand-engineered features (Fig. S9) near the image center, (ii) task-specific features from deep learning throughout the image, (iii) whether IHC or other markers were mentioned for this case, and (iv) optionally tissue type. Other machine learning tasks, e.g., histology stain prediction and tissue type prediction, were simpler. For simpler tasks, we used only the Random Forest and 2412 hand-engineered features, without deep learning.

We train a Random Forest of 1000 trees as a baseline for all tasks. A white-balanced image is scaled so its shortest dimension is 512 pixels (px). White balancing helps correct images with reduced blue coloration due to low lighting (Fig. S5D). The 512 × 512 px center crop is then extracted, and 2412 hand-engineered image features are calculated for this crop (Figs. 3 and S9).

### Customized hybrid deep-learning-Random-Forest model and clinical covariates

#### Image preprocessing and data augmentation

For image preprocessing, a white-balanced image is scaled to be 512 px in its shortest dimension, and for deep learning, 224 × 224 px patches are sampled to train a deep convolutional neural network. For deep learning, we use data augmentation of random rotations, random flips, random zoom/rescaling, random brightness variations, Gaussian noise, and Mixup [28]. This means that throughout training hundreds of times over our data we make many small changes to the data each time, e.g., to teach the neural network that rotating an image does not change the diagnosis. “Deep learning” (Section S5.11.1) in the supplement discusses further.

#### Deep learning and deep features

To maximize performance by learning disease-state-specific features, we additionally consider deep learning for the most challenging task of disease state prediction. Our deep learning architecture is a ResNet-50 [29] (Fig. 3B) pretrained on ImageNet, which we train end-to-end without freezing layers (Fig. S13). This means the ResNet-50 deep convolutional neural network is initially trained to classify natural images, e.g., cats and dogs, but every neuron may be adjusted in a data-driven manner for histology-specific learning on our pathology Twitter dataset. To determine how deep feature representations change before and after training the ResNet-50 on histopathology images and covariates, we analyze both (i) ImageNet_2048_ features from the ResNet-50 that has not been trained on histopathology data, and (ii) 100 deep features based on the same ResNet-50 where all neurons have been further trained on histopathology data. We define ImageNet_2048_ features as the 2048 outputs from the ResNet-50’s final Global Average Pooling layer, summed over 21 image patches in a grid fashion and concatenated with other features for Random Forest learning (Fig. 3C). For histopathology deep learning, we append a 100-neuron fully-connected layer atop the ResNet-50, connecting to the ResNet-50 and covariates, and sum over the same 21 image patches in a grid fashion (Fig. 3B). “Deep learning instance and set feature vectors” (Section S5.8.1) in the supplement discusses this and the feature interpretability related to the Heaviside step function (Eqs. 6 and 8).

#### Clinical covariates

To best predict disease state and find similar cases, we seek to include as much patient-related context as possible in our computational pathology machine learning models, so we additionally include clinical information, i.e., tissue type and marker mentions. To represent the tissue type covariate, we include a ten-dimensional one-hot-encoded binary vector to encode which one of the ten possible tissue types is present for this case. If the tissue type is unknown, tissue type is all zeroes for the neural network while being missing values for the Random Forest. We also include a binary one-dimensional marker mention covariate, which is 1 if any pathologist discussing the case mentions a marker test, e.g., “IHC” or “desmin”.

### Disease state classifier repurposed for similarity-based search

After we train a Random Forest classifier (see “Random Forest classifier” in “Materials and methods”) to predict/classify disease state from a variety of deep and non-deep features (Fig. 3C), we then use this classifier’s Random Forest similarity metric for search [30, 31]. Specifically, our Random Forest consists of 1000 Random Trees, each of which predicts disease state. If any given Random Tree makes an identical sequence of decisions to classify two histopathology images (each with optionally associated clinical covariates), the similarity of those two images is incremented by one. Aggregating across all Random Trees, the similarity of any two images can therefore be quantified as a number between 0 (not similar according to any Random Tree) and 1000 (similar according to all 1000 Random Trees). Equipped with this similarity metric, we repurpose the classifier for search: the classifier takes in a search image and compares it to each other image using this similarity metric, then provides a list of images ranked by similarity to the search image. This approach provides the first pan-tissue (i.e., bone and soft tissue, breast, dermatological, gastrointestinal, genitourinary, gynecological, head and neck, hematological, neurological, pulmonary, etc.) pan-disease (i.e., nontumor, low grade, and malignant) case search in pathology.

### Three levels of sanity checking for search

To inform the physician and to avoid mistakes, sanity checks are important in medicine, or wherever human life may be at risk. Quantifying uncertainty is particularly important [25] in medicine, to assess how much trust to put in predictions that will affect the patient’s care. We are the first to offer three sanity checks for each individual search: (i) prediction uncertainty, (ii) prediction as a check for search, and (iii) prediction heatmaps. “Machine learning sanity checking for search” discusses further (Section S5.9). Briefly, “prediction uncertainty” relies on an ensemble of classifiers to assess if prediction strength is statistically significant, and if not, the prediction and search using this image should not be trusted. Second, “prediction as a check for search” indicates that if the disease state classification for a given image is assessed as incorrect by a pathologist, search results using this image should not be trusted, because the same classifier is used for both prediction and search. Third, we use “prediction heatmaps” to show disease state predictions for each component of a given image, based on deep learning. If a pathologist disagrees with these heatmaps, deep-learning-based search for that image cannot be trusted. A failure of any one of these three checks indicates that search results may be incorrect, and they are flagged as such.

### Five levels of method interpretability

Interpretability is critical in medicine [24] for physicians to understand whether or not the machine learning is misinterpreting the data. For example, machine learning may uncover that pneumonia patients with a history of asthma have lower mortality risk, suggesting that asthma is protective against pneumonia mortality. However, this would not make sense to a physician, who would instead realize that such patients have lower mortality because they are more likely to be admitted directly to an intensive care unit [32, 33]. Asthma is not protective from pneumonia mortality, intensive care is.

**Fig. 4:**
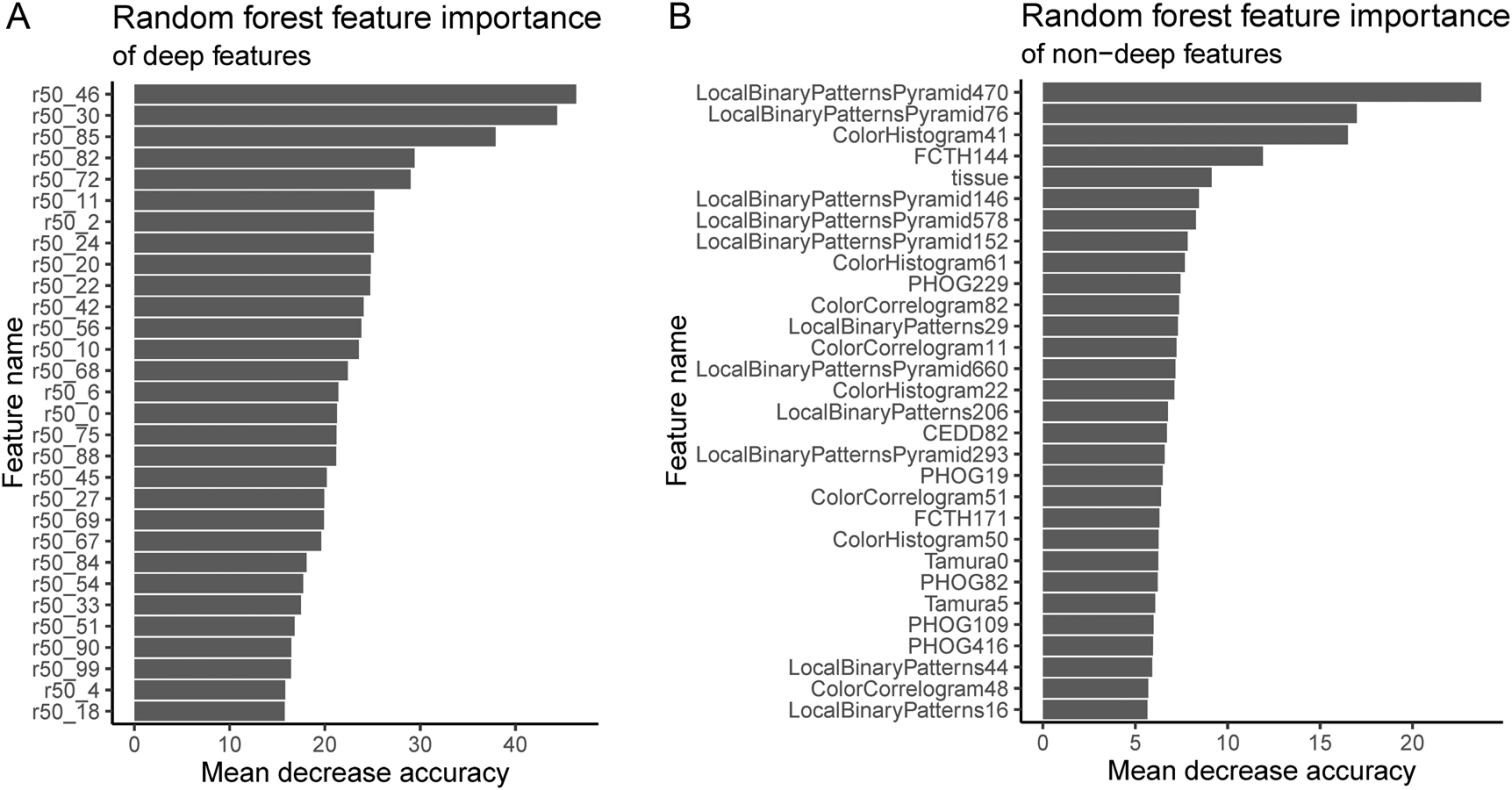
Random Forest feature importance for prioritizing deep features, when non-deep, deep, and clinical features are used together for learning. We use the mean decrease in accuracy to measure Random Forest feature importance. To do this, first, a Random Forest is trained on task-agnostic hand-engineered features (e.g., color histograms), task-specific deep features (i.e., from the ResNet-50), and the tissue type covariate that may be missing for some patients. Second, to measure the importance of a feature, we randomly permute/shuffle the feature’s values, then report the Random Forest’s decrease in accuracy. When shuffling a feature’s values this way, more important features result in a greater decrease in accuracy, because accurate prediction relies on these features more. We show the most important features at the top of these plots, in decreasing order of importance, for deep features (**A**) and non-deep features (**B**). The most important deep feature is “r50_46”, which is the output of neuron 47 of 100 (first neuron is 0, last is 99), in the 100-neuron layer we append to the ResNet-50. Thus of all 100 deep features, r50_46 may be prioritized first for interpretation. Of non-deep features, the most important features include Local Binary Patterns Pyramid (LBPP), color histograms, and “tissue” (the tissue type covariate). LBPP and color histograms are visual features, while tissue type is a clinical covariate. LBPP are pyramid-based grayscale texture features that are scale-invariant and color-invariant. LBPP features may be important because we neither control the magnification a pathologist uses for a pathology photo, nor do we control. staining protocol. For a before-and-after training comparison that may suggest the histopathology-trained deep features represent edges, colors, and tissue type rather than texture, we also analyze feature importance of only natural-image-trained ImageNet_2048_ deep features in conjunction with hand-engineered features (Fig. S10). “Marker mention and SIFT features excluded from Random Forest feature importance analysis” discusses other details in the supplement (Section S5.10.2).

**Fig. 5:**
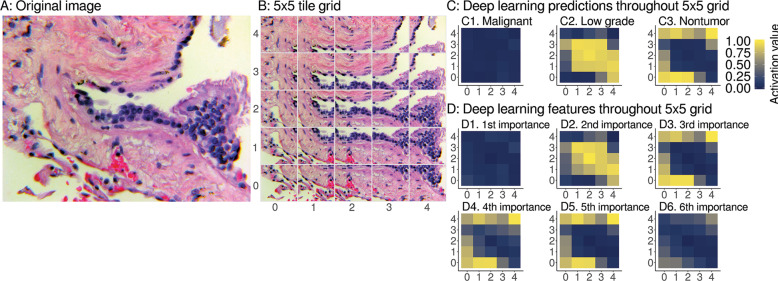
Interpretable spatial distribution of deep learning predictions and features. **A** An example image for deep learning prediction interpretation, specifically a pulmonary vein lined by enlarged hyperplastic cells, which we consider to be low-grade disease state. Case provided by YR. **B** The image is tiled into a 5 × 5 grid of overlapping 224 × 224 px image patches. For heatmaps, we use the same 5 × 5 grid as in Fig. 1C bottom left, imputing with the median of the four nearest neighbors for 4 of 25 grid tiles. **C** We show deep learning predictions for disease state of image patches. At left throughout the image, predictions have a weak activation value of 0 for malignant, so these patches are not predicted to be malignant. At middle the centermost patches have a strong activation value of 1, so these patches are predicted to be low grade. This spatial localization highlights the hyperplastic cells as low grade. At right the remaining normal tissue and background patches are predicted to be nontumor disease state. Due to our use of softmax, we note that the sum of malignant, low-grade, and nontumor prediction activation values for a patch equals 1, like probabilities sum to 1, but our predictions are not Gaussian-distributed probabilities. **D** We apply the same heatmap approach to interpret our ResNet-50 deep features as well. **D1** the most important deep feature corresponds to the majority class prediction, i.e., **C1**, malignant. **D2** The second most important deep feature corresponds to prediction of the second most abundant class, i.e., **C2**, low grade. **D3** The third most important deep feature corresponds to prediction of the third most abundant class, i.e., **C3**, nontumor. The fourth (**D4**) and fifth (**D5**) most important features also correspond to nontumor. **D6** The sixth most important deep feature does not have a clear correspondence when we interpret the deep learning for this case and other cases (Fig. S11), so we stop interpretation here. As expected, we did not find ImageNet_2048_ features to be interpretable from heatmaps, because these are not trained on histpathology (Fig. S11A5).

**Fig. 6:**
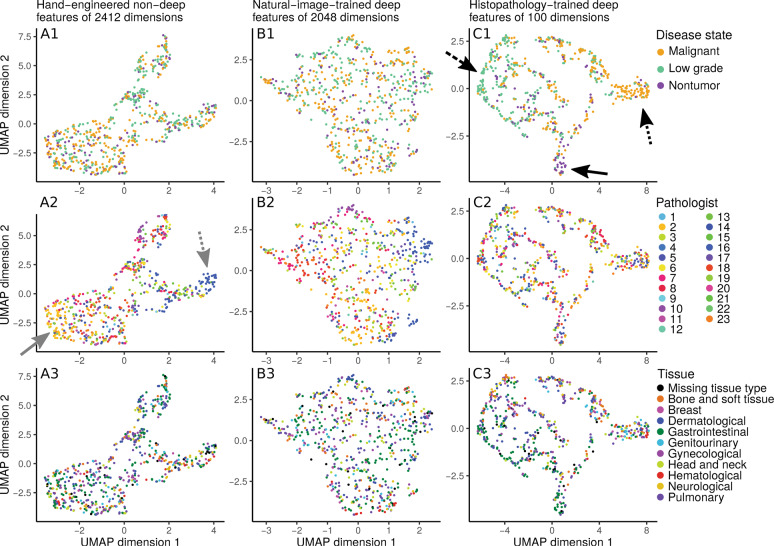
Disease state clusters based on hand-engineered, natural-image-trained deep features, or histopathology-trained deep features. To determine which features meaningfully group patients together, we apply the UMAP [34] clustering algorithm on a held-out set of 10% of our disease state data. Each dot represents an image from a patient case. In general, two dots close together means these two images have similar features. Columns indicate the features used for clustering: hand-engineered features (at left column), only-image-trained ImageNet_2048_ deep features (at middle column), or histopathology-trained deep features (at right column). Rows indicate how dots are colored: by disease state (at top row), by contributing pathologist (at middle row), or by tissue type (at bottom row). For hand-engineered features, regardless of whether patient cases are labeled by disease state (**A1**), pathologist (**A2**), or tissue type (**A3**), there is no strong clustering of like-labeled cases. Similarly, for only natural-image-trained ImageNet_2048_ deep features, there is no obvious clustering by disease state (**B1**), pathologist (**B2**), or tissue type (**B3**). However, for histopathology-trained deep features, patient cases cluster by disease state (**C1**), with separation of malignant (at dotted arrow), low grade (at dashed arrow), and nontumor (at solid arrow). There is no clear clustering by pathologist (**C2**) or tissue type (**C3**). The main text notes that hand-engineered features may vaguely group by pathologist (**A2**, pathologists 2 and 16 at solid and dotted arrows).

Ideally, interpretability facilitates both deductive and inductive human reasoning about the machine learning findings. Deductively, interpretability allows human reasoning about what machine learning finds in specific patient cases, e.g., explaining the malignant prediction overall for a patient by spatially localizing malignancy-related feature activations. Inductively, interpretability allows human reasoning about broad principles that may be inferred from the machine learning findings overall for a task, e.g., texture importance in disease state prediction. To the best of our knowledge, it is novel to offer both in a pan-tissue pan-disease manner in computational pathology. We do this with (i) hand-engineered feature interpretability (Fig. S9), (ii) Random Forest feature importance (Fig. 4), (iii) before-and-after histopathology-training feature importance comparison of deep features to hand-engineered features (Fig. 4 vs. Fig. S10), (iv) deep feature activation maps (Figs. 5D and S11), and (v) cluster analyses (Fig. 6). “Machine learning interpretability for search” in the supplement discusses further (Section S5.10).

#### Histopathology-trained deep features represent edges, colors, and tissue

To understand what deep features learn to represent after training on histopathology data, we compare Random Forest feature importances of (a) ImageNet 2048 deep features [not trained on histopathology data] with hand-engineered features and tissue covariate (Fig. S10), to (b) 100 deep features [trained on histopathology data] with hand-engineered features and tissue covariate (Fig. 4). Before the deep neural network is trained on histopathology data, the tissue covariate as well as edge and color hand-engineered features are important (Fig. S10). However, after the deep neural network is trained on histopathology data, tissue is less important while texture hand-engineered features are more important (Fig. 4). Therefore, we reason that the deep neural network learns histopathology-relevant edge, color, and tissue features from histopathology data (which reduces the importance of e.g., hand-engineered edge and color features after learning), but the deep neural network may forget histopathology-relevant texture features during learning (which increases the importance of hand-engineered texture features after learning).

#### Interpretability uncovers spatial prediction-to-feature correspondences of disease

Considering both introspective/inductive interpretability (Fig. 4) and demonstrative/deductive interpretability (Fig. 5), we find a correspondence between important deep features (Fig. 4) and the spatial localization of deep learning predictions (Fig. 5). We find that using (Eq. 14) the three most important interpretable deep features slightly but significantly improve search performance (Table S1). “Deep set learning feature interpretation” discusses further (Section S5.11.2).

#### Deep features trained on histopathology logically cluster patients by disease state, whereas pathology-agnostic features do not

Through cluster analysis we interpret which features (i.e., hand-engineered, only natural-image-trained, or histopathology-trained), if any, separate patients into meaningful groups, and if the features “make sense” to describe patient histopathology. As expected, neither hand-engineered features (Fig. 6A1) nor only natural-image-trained ImageNet_2048_ deep features (Fig. 6B1) cluster patient cases by disease state, presumably because these features are not based on histopathology. These approaches also do not cluster patients by contributing pathologist (Fig. 6A2, B2) or by tissue type (Fig. 6A3, B3). In addition, we do not find that reducing dimensionality through principal components analysis qualitatively changes the clustering (Fig. S12). In contrast, deep features trained on histopathology data do cluster patients together by disease state (Fig. 6C1), but not by pathologist (Fig. 6C2) or tissue (Fig. 6C3). We conclude that these deep features primarily reflect representations of disease state in a non-tissue-specific manner. It is important to note that any clustering-based result must be carefully scrutinized, because features may suffer from artifacts, e.g., which pathologist shared the patient case. If taken to an extreme, learning to predict disease state on the basis of pathologist-specific staining/lighting/camera artifacts amounts to learning concepts such as, “if pathologist X typically shares images of malignant cases, and a new image appears to be from pathologist X, then this image probably shows malignancy”, which does not “make sense” as a way to predict disease state. Although we did not observe robust clustering by pathologist, even vague grouping by pathologist (Fig. 6A2 at gray arrows) highlights the importance of critically assessing results. Artifact learning risk is one reason why we (i) rigorously test search through leave-one-pathologist-out cross-validation, and (ii) provide sanity checks.

### Experimental design and evaluation

We evaluate our classifiers using ten-fold cross-validation to estimate bounds of accuracy and AUROC performance metrics. “Supplementary experimental design and evaluation” explains further (Section S5.14). Because we intend for our methods to accurately find similar cases for any pathologist worldwide, we rigorously test search using leave-one-pathologist-out cross-validation and report precision@k. Leave-one-pathologist-out cross-validation isolates pathologist cases from one another, so a test set is independent from the corresponding training set. This isolates to a test set pathologist-specific or institution-specific imaging artifacts that may occur from microscopy, lighting, camera, or staining protocol. Thus our leave-one-pathologist-out cross-validation measurements quantify our method’s reproducibility, which is critical to measure in medical machine learning [7].

### Social media bot for public prospective testing

We present the first pathology-specific social media bot, @pathobot, on Twitter. This bot is a case similarity search tool that applies our methods. Pathologists on Twitter mention the bot in a tweet containing an image. The bot uses our Random Forest classifier to provide disease state prediction for that image, and search for similar results. Its prediction and search results, along with quantitative assessments of prediction uncertainty, are provided to pathologists in real time. In this way, the bot facilitates prospective tests, and encourages collaboration: as pathologists use the bot, they provide us with complementary qualitative feedback and help us recruit additional collaborators. In this way, the bot facilitates prospective tests, and encourages collaboration: as pathologists publicly use the bot, they provide us with complementary qualitative feedback and these interactions help us recruit additional collaborators.

### Computational hardware

For machine learning, we use Weka version 3.8.1 [35] on a laptop. For deep learning, we use Tensorflow version 1.0.0 with Keras version 2.1.4 [36] on a supercomputing cluster having GPUs supporting nVidia CUDA version 8.0 and cuDNN version 5.1. “Supplementary computational hardware and software” discusses further (Section S5.15). In R, we perform feature importance analyses with the randomForest package [37] and cluster analyses with the umap package [38].

## Results

### Identifying and filtering for H&E images

We ran increasingly difficult tests using increasingly sophisticated machine learning methods. Our first question is the most basic, but arguably the most important: can machine learning distinguish acceptable H&E-stained human pathology images from all others (Figs. 2A, S4, and S5)? We show acceptable H&E-stained human pathology images can be distinguished from other images—e.g., natural scenes or different histochemistry stains (Fig. 6 at left) with high performance (AUROC 0.95). Because of the high performance of this classifier, it can be used to partially automate one of our manual data curation tasks, i.e., identifying acceptable images on social media. More importantly, when confronted with over one million PubMed articles, we apply this classifier to filter out all the articles that do not have at least one H&E image. To our knowledge, this is the first H&E image detector to filter PubMed articles. PubMed figures increase our searchable dataset by over an order of magnitude, without any additional manual curation effort. Only with a large dataset may we expect to successfully search for rare diseases, and we currently have 126,787 searchable images. This task also serves as a positive control.

### Distinguishing common stain types

**Fig. 7:**
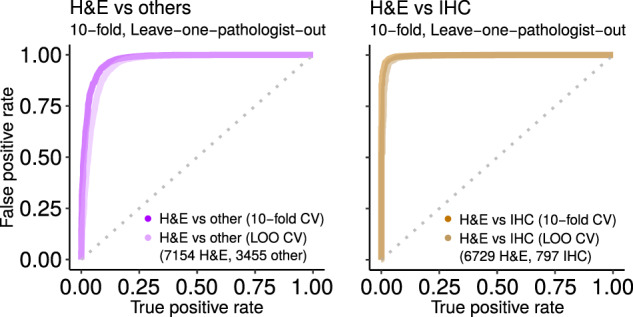
H&E performance. Predicting if an image is acceptable H&E human tissue or not (at left), or if image is H&E rather than IHC (at right). Ten replicates of ten-fold cross-validation (tenfold) and leave-one-pathologist-out cross-validation (LOO) had similarly strong performance. This suggests the classifier may generalize well to other datasets. We use the “H&E vs. others” classifier to find H&E images in PubMed. Shown replicate AUROC for H&E vs. others is 0.9735 for tenfold (ten replicates of tenfold has mean ± stdev of 0.9746 ± 0.0043) and 0.9549 for LOO (ten replicates 0.9547 ± 0.0002), while H&E vs. IHC is 0.9967 for tenfold (ten replicates 0.9977 ± 0.0017) and 0.9907 for LOO (ten replicates 0.9954 ± 0.0004). For this and other figures, we show the first replicate.

H&E and IHC stain types are the most common in our dataset and are common in practice. We therefore ask if machine learning can distinguish between these stain types, which vary in coloration (Fig. 2A). Indeed, the classifier performs very well at this discrimination (AUROC 0.99, Fig. 7 at right). Thus, although IHC coloration can vary between red and brown, machine learning can still successfully differentiate it from H&E. “Intra-stain diversity” explains further (Section S5.3.1). A well-performing classifier such as this can be useful with large digital slide archives that contain a mixture of H&E and IHC slides that lack explicit labels for staining information. Our classifier can automatically and accurately distinguish these stains, so that downstream pipelines may process each stain type in a distinct manner.

### Distinguishing ten histopathology tissue types

**Fig. 8:**
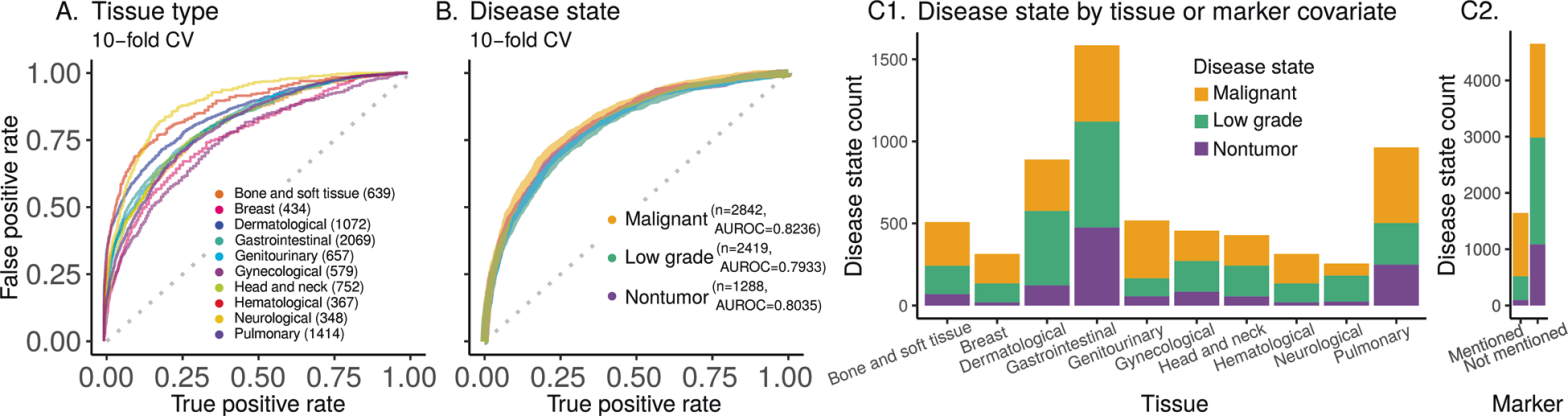
Ten tissue type and three disease state prediction performance and counts. **A** Classifier performance for predicting histopathology tissue type (ten types, 8331 images). **B** Classifier performance for predicting disease state (three disease states; 6549 images). Overall AUROC is the weighted average of AUROC for each class, weighted by the instance count in the class. These panels (**A**, **B**) show AUROC (with ten-fold cross-validation) for the chosen classifier. Random Forest AUROC for tissue type prediction is 0.8133 (AUROC for the ten replicates: mean ± stdev of 0.8134 ± 0.0007). AUROC is 0.8085 for an ensemble of our deep-learning-Random-Forest hybrid classifiers for disease state prediction (AUROC for the ten replicates: mean ± stdev of 0.8035 ± 0.0043). **C1** Disease state counts per tissue type. The proportion of nontumor vs. low-grade vs. malignant disease states varies as a function of tissue type. For example, dermatological tissue images on social media are most often low grade, but malignancy is most common for genitourinary images. **C2** Disease state counts as a function of whether a marker test (e.g., IHC, FISH) was mentioned (˜25% of cases) or not. IHC is the most common marker discussed and is typically, but not necessarily, used to subtype malignancies.

We next ask if machine learning can distinguish the ten tissue types present in our Twitter dataset (Fig. 2A). “Tissue hashtags and keywords” discusses this further (Section S5.6.2). The tissue types were distinguishable (AUROC 0.81, Fig. 8A) and, as expected, this task was more difficult than stain-related tasks. Being able to identify tissue types may help the detection of contaminating tissue in a slide.

### Deep learning predicts disease state across many tissue types

**Fig. 9:**
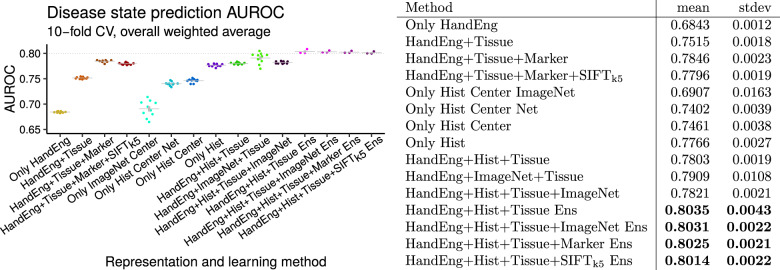
Disease state prediction performance for machine learning methods.. For deep learning we use a ResNet-50. For shallow learning we use a Random Forest. We train a Random Forest on deep features (and other features), to combine deep and shallow learning (Fig. 3C top). Error bars indicate standard error of the mean. Points indicate replicates. Gray lines indicate means. Performance increases markedly when including tissue type covariate for learning (even though tissue type is missing for some patients), when using deep learning to integrate information throughout entire image rather than only the center crop, and when using an ensemble of classifiers. Performance exceeds AUROC of 0.8 (at right). We conclude method xii (“HandEng + Hist + Tissue Ens”) is the best we tested for disease state prediction, because no other method performs significantly better and no other simpler method performs similarly. Methods are, from left to right, (i) Random Forest with 2412 hand-engineered features alone for 512 × 512 px scaled and cropped center patch, (ii) Random Forest with tissue covariates, (iii) Random Forest with tissue and marker covariates, (iv) method iii additionally with SIFT_k5_ features for Random Forest, (v) only natural-image-trained ResNet-50 at same scale as method i with center 224 × 224 px center patch and prediction from a Random Forest trained on 2048 features from the ResNet-50 (Fig. 3), (vi) histopathology-trained ResNet-50 at same scale as method i with center 224 × 224 px center patch and prediction from top three neurons (Fig. 3B top), (vii) histopathology-trained ResNet-50 with Random Forest trained on 100 features from 224 × 224 px center patch per method vi, (viii) histopathology-trained ResNet-50 features at 21 locations throughout image summed and Random Forest learning on this 100-dimensional set representation with 2412 hand-engineered features, (ix) method viii with tissue covariates for histopathology-trained ResNet-50 and 2412 hand-engineered features for Random Forest learning (i.e., Fig. 3C sans marker information), (x) method ix with an only natural-image-trained ResNet-50 instead of a histopathology-trained ResNet-50 for Random Forest learning, (xi) method ix with both an only natural-image-trained ResNet-50 and a histopathology-trained ResNet-50 for Random Forest learning, (xii) method ix with an ensemble of three Random Forest classifiers such that each classifier considers an independent histopathology-trained ResNet-50 feature vector in addition to 2412 hand-engineered features and tissue covariate, (xiii) method xii where each Random Forest classifier in ensemble additionally considers only natural-image-trained ResNet-50 features, (xiv) method xii where each Random Forest classifier in ensemble additionally considers the marker mention covariate (i.e., this is an ensemble of three classifiers where Fig. 3C is one of the three classifiers), (xv) method xii where each Random Forest in ensemble additionally considers SIFT_k5_ features for learning.

Pathologists routinely make decisions about whether a tissue shows evidence of nontumoral disease, low-grade disease, or malignant disease, while ignoring spurious artifacts (Fig. S1). We therefore ask whether machine learning can perform well on this clinically important task. For this, we use our most common stain type, H&E, including only those images that are single-panel and deemed acceptable (Fig. S4). We systematically test increasingly sophisticated machine learning methods (Fig. 9) with the goal of achieving the highest possible performance. The simplest baseline model we consider, a Random Forest on the 2412 hand-engineered features (Fig. S9), achieves an AUROC of 0.6843 ± 0.0012 (mean ± stdev, Fig. 9). Conversely, an ensemble of our deep-learning-Random-Forest hybrid classifiers achieves much higher performance, with AUROC 0.80 (Fig. 9). To our knowledge, this is the first classifier that predicts the full spectrum of disease states, i.e., nontumor, low grade, and malignant (Figs. 2, 8B, and 9).

### Texture and tissue are important clinico-visual features of disease

We next determine which features are important to our machine learning classifier for disease state prediction. To do this, we interpret the Random Forest feature importance to gain insight into the clinico-visual features that are predictive of disease state. Our analyses suggest that texture (e.g., local binary patterns) and color (e.g., color histograms) features are most important for pathology predictions and search, followed by the tissue type clinical covariate (Fig. 4). “Marker mention and SIFT features excluded from Random Forest feature importance analysis” discusses further (Section S5.10.2). Our method is therefore multimodal, in that it learns from both visual information in the images and their associated clinical covariates (e.g., tissue type and marker mention). Both modalities improve search performance, as discussed in the following section.

### Disease state search, first pan-tissue pan-disease method

**Fig. 10:**
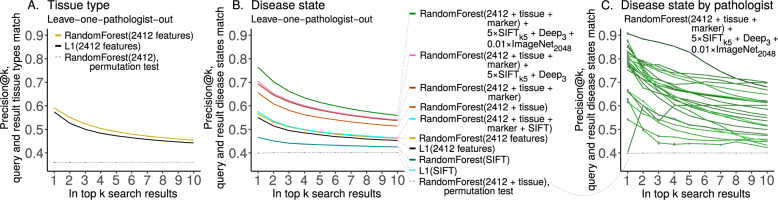
Case similarity search performance. We report search performance as precision@*k* for leave-one-pathologist-out cross-validation for **(A)** tissue and **(B)** disease state. We note search based on SIFT features performs better than chance, but worse than all alternatives we tried. Marker mention information improves search slightly, and we suspect cases that mention markers may be more relevant search results if a query case also mentions markers. SIFT_k5_ and histopathology-trained Deep_3_ features improve performance even less, but only natural-image-trained ImageNet_2048_ deep features increase performance substantially (Table S1). **(C)** We show per-pathologist variability in search, with outliers for both strong and weak performance. Random chance is dashed gray line. In our testing, performance for every pathologist is always above chance, which may suggest performance will be above chance for patient cases from other pathologists. We suspect variability in staining protocol, variability in photography, and variability in per-pathologist shared case diagnosis difficulty may underlie this search performance variability. The pathologist where precision@*k* = 1 is lowest shared five images total for the disease prediction task, and these images are of a rare tissue type. Table S2 shows per-pathologist performance statistics.

In light of pathology-agnostic approaches to pathology search [18, 19], we ask if pathology-specific approaches to pathology search may perform better. Indeed, search is the main purpose of our social media bot. Moreover, others have noted task-agnostic features may suffer from poorly understood biases, e.g., features to distinguish major categories (e.g., cats and dogs) in natural images may systematically fail to distinguish major categories in medical images (e.g., ophthalmology or pathology) [39]. To evaluate performance of search, we show precision@*k* for *k* = 1,…, 10 (Fig. 10). As a positive control, we first test search for similar tissues (Fig. 9A), e.g., if the search query image is breast pathology then the top search results should be breast pathology. Here, precision@*k* = 1 = 0.6 means 60% of the time the search query image and top search result image have matching tissue types, e.g., both are breast, or both are gastrointestinal, etc. We subsequently test search for similar disease states (Fig. 9B and Table S1), e.g., if the search query image is malignant then the top search results should be malignant. Here, precision@*k* = 1 = 0.76 means 76% of the time the search query image and top search result image have matching disease states (e.g., both malignant, both nontumor, etc), while precision@*k* = 8 = 0.57 means the search query image matches 57% of the top eight search results, i.e., 4–5 of the top eight search results are malignant when the search query image is malignant. To estimate performance in general for each method, we perform ten replicates of leave-one-pathologist-out cross-validation with different random seeds (i.e., 0, 1, …, 9). This allows variance to be estimated for Random Forest learning, but methods based exclusively on the L1 norm are fully deterministic, so these have zero estimated variance (Table S1). We follow two-sample hypothesis testing, where one set of ten replicates is compared with a different set of ten replicates. To calculate a *U* statistic and a *p* value, we use the two-tailed Wilcoxon rank-sum test on precision@*k* = 1, which tests for significant differences in precision for the first search result on average. For search’s statistical null model, we train a Random Forest on images with randomly shuffled class labels and evaluate precision@*k*, as a permutation test (i.e., “RandomForest(2412 + tissue), permutation test” precision@*k* = 1 = 0.3967 ± 0.0044 in Table S1, shown in Fig. 9B). We conclude search performs significantly better than chance (0.7618 ± 0.0018 vs 0.3967 ± 0.0044, *U* = 100, *p* = 0.0001817) and offer specifics below.

Results for disease state search are detailed in “supplementary disease state search results” (Section S5.13). Here, we briefly describe three main findings. First, “clinical covariates improve search performance” (Section S5.13.1). Both tissue type (0.5640 ± 0.0024 vs. 0.6533 ± 0.0025, *U* = 100, *p* = 0.0001796) and marker mention (0.6533 ± 0.0025 vs. 0.6908 ± 0.0021, *U* = 100, *p* = 0.0001796) covariates significantly improve search performance. This suggests that for search these clinical features provide disease state information above and beyond the visual characteristics we have of each image. Second, “in the context of other features, nuclear features of disease are better represented by the most prevalent SIFT clusters rather than all SIFT” (Section S5.13.2), and the effect of scale-invariant feature transform (SIFT) clusters on search is small but significant (0.6908 ± 0.0021 vs. 0.6935 ± 0.0029, *U* = 19.5, *p* = 0.02308). This indicates nuclear features, as represented by SIFT, provide limited but complementary disease-related information for search. Third, “deep features synergize with other features, informing search more than nuclear SIFT features, but less than clinical covariates” (Section S5.13.3). Specifically, deep features improve search performance less than tissue type (0.5720 ± 0.0036 vs. 0.6533 ± 0.0025, *U* = 0, *p* = 0.0001806) and less than marker mentions (0.6602 ± 0.0022 vs. 0.6908 ± 0.0021, *U* = 0, *p* = 0.0001817), but more than SIFT clusters (0.6983 ± 0.0016 vs. 0.6948 ± 0.0032, *U* = 83.5, *p* = 0.01251). Fourth, “deep features trained only on natural images outperform hand-engineered features for search, and offer best performance when combined with other features” (Section S5.13.4). Particularly, in the context of clinical covariates, ImageNet_2048_ features demonstrate high importance by offering better search performance than the 2412 hand-engineered features, SIFT_k5_ features, and histopathology-trained Deep_3_ features *combined* (0.7517 ± 0.0025 vs. 0.7006 ± 0.0026, *U* = 100, *p* = 0.0001817)—although this may change as more data become available or more advanced methods are used. Moreover, we found that adding only natural-image-trained ImageNet_2048_ deep features to our best-performing model (incorporating hand-engineered features, tissue type, marker mention, SIFT_k5_ features, and Deep_3_ features) improved search performance further (0.7006 ± 0.0026 vs. 0.7618 ± 0.0018, *U* = 0, *p* = 0.0001817), and was the best-performing search method we measured. Taken together, we conclude (i) texture and tissue features are important, (ii) histopathology-trained deep features are less important, (iii) nuclear/SIFT features are least important for disease state search, and (iv) in the context of clinical covariates the only-natural-image-trained ImageNet_2048_ deep features are the most important visual features we tested for search.

## Discussion

### Summary

Pathologists worldwide reach to social media for opinions, often sharing rare or unusual cases, but replies may not be immediate, and browsing potentially years of case history to find a similar case can be a time-consuming endeavor. Therefore, we implemented a social media bot that in real-time searches for similar cases, links to these cases, and notifies pathologists who shared the cases, to encourage discussion. To facilitate disease prediction and search, we maintain a large pathology-focused dataset of 126,787 images with associated text, from pathologists and patients the world over. This is the first pan-tissue, pan-disease dataset in pathology, which we will share with the community through pathobotology.org to promote novel insights in computational pathology. After performing stain- and tissue-related baselines with a Random Forest, we performed a number of analyses on this dataset for disease state prediction and search. To accomplish this, we developed a novel synthesis of a deep convolutional neural network for image set representations and a Random Forest learning from these representations (Fig. 3 and S14). We found this model can classify disease state with high accuracy, and be repurposed for real-time search of similar disease states on social media. This interpretable model, combined with its social media interface, facilitates diagnoses and decisions about next steps in patient care by connecting pathologists all over the world, searching for similar cases, and generating predictions about disease states in shared images. Our approach also allowed us to make a number of important methodological advances and discoveries. For example, we found that both image texture and tissue are important clinico-visual features of disease state—motivating the inclusion of both of feature types in multimodal methods such as ours. In contrast, deep features and cell nuclei features were less important for search. Finally, we provide important technical advances, because our novel deep feature regularization and activation functions yield approximately binary features and set representations that may be applicable to other domains. In sum, these advances readily translate to patient care by taking advantage of cutting-edge machine learning approaches, large and diverse datasets, and interactions with pathologists worldwide.

### Comparison with prior studies

Our approach builds on, but greatly extends, prior work in the field of computational pathology. We will comment on this briefly here, and describe more fully in “supplementary comparison with prior studies” (Section S5.16). First, much of prior work involves a subset of tissue types or disease states [40–42]. However, our study encompasses diverse examples of each. Second, prior studies investigating pathology search take a variety of pathology-agnostic approaches, e.g., (i) using neural networks that were not trained with pathology data [18, 19] or (ii) using SIFT features [19, 43, 44] that do not represent texture or color [45]. Our inclusive approach is different, training a model on pathology data represented by thousands of features—including SIFT clusters, neural networks, other visual features, and clinical covariates. Our model outperforms pathology-agnostic baselines.

Prior work has found texture and/or color to be important for tissue-related tasks in computational pathology [46–48]. We find texture and color to be important for disease-related tasks. In addition, we go a step further by comprehensively considering the relative contributions of many clinico-visual features to the prediction and search of disease. Such important features include texture, color, tissue type, marker mentions, deep features, and SIFT clusters.

### Caveats and future directions

Below we discuss the primary caveats (also see “supplementary caveats” in Section S5.17) and future directions (also see “supplementary future directions” in Section S5.18).

#### Diagnosis disagreement or inaccuracy

First, there is a risk of error in our data because many different pathologists share cases, and they may disagree on the most appropriate hashtags or diagnosis. Moreover, there may be diagnostic inaccuracies from the pathologist who posted the case, or other pathologists. We find these situations to be rare, but if they occur, the case tends to have an increased amount of discussion, so we can identify these situations. Second, our nontumor/low-grade/malignant keyword rules may be incorrect or vague. For these first and second caveats, we take a majority vote approach, manually curate as needed, and discuss. Indeed, as we discussed amongst ourselves the hyperplasia in Fig. 5, it became clear we needed to explicitly mention preneoplastic disease is included in the low-grade disease state category.

#### Dataset case sampling and region of interest biases

Our dataset may have both (i) a case sampling bias and (ii) a region of interest sampling bias. First, there may be case sampling bias if we typically have unusual cases that pathologists consider worth sharing, and our cases by necessity only come from pathologists on social media. We plan to advocate sharing of normal tissue and less unusual cases to circumvent this bias. Second, the pathologist who shares the case chooses which images to share, typically sharing images of regions of interest that best illustrate the diagnosis, while ignoring other slides where the diagnosis is less straightforward. In future work, we will include whole slide images for additional context.

#### Dataset size and granularity

To increase the granularity and accuracy of tissue-type predictions, we first plan to expand the size of this dataset by recruiting more pathologists via social media, aiming to have representative images for each organ. There are many organs within the gastrointestinal tissue type, for instance. In additiony, we expect our dataset to broaden, including more social media networks and public pathology resources such as TCGA, with our bot integrating these data for search and predictions.

## Conclusion

We believe this is the first use of social media data for pathology case search and the first pathology study prospectively tested in full public view on social media. Combining machine learning for search with responsive pathologists worldwide on social media, we expect our project to cultivate a more connected world of physicians and improve patient care worldwide. We invite pathologists and data scientists alike to collaborate with us to help this nascent project grow.

## Supplementary information

supplementary text and figures
